# Decentralized Coordination of a Multi-UAV System for Spatial Planar Shape Formations

**DOI:** 10.3390/s23239553

**Published:** 2023-12-01

**Authors:** Etienne Petitprez, François Guérin, Frédéric Guinand, Florian Germain, Nicolas Kerthe

**Affiliations:** 1 Le Havre Normandy University, 76600 Le Havre, France; etienne.petitprez@squadrone-system.com (E.P.); frederic.guinand@univ-lehavre.fr (F.G.); florian.germain@univ-lehavre.fr (F.G.); nicolas.kerthe@univ-lehavre.fr (N.K.); 2 Squadrone System, 38000 Grenoble, France; 3 LITIS Laboratory, 76600 Le Havre, France; 4 GREAH Laboratory, 76600 Le Havre, France; 5 Faculty of Mathematics and Natural Sciences, Cardinal Stefan Wyszynski University, 01-815 Warsaw, Poland

**Keywords:** multi-UAVs, decentralized coordination, Fourier’s descriptors, formation control, pattern formation, collision avoidance

## Abstract

Motivated by feedback from firefighters in Normandy, this work aims to provide a simple technique for a set of identical drones to collectively describe an arbitrary planar virtual shape in a 3D space in a decentralized manner. The original problem involved surrounding a toxic cloud to monitor its composition and short-term evolution. In the present work, the pattern is described using Fourier descriptors, a convenient mathematical formulation for that purpose. Starting from a reference point, which can be the center of a fire, Fourier descriptors allow for more precise description of a shape as the number of harmonics increases. This pattern needs to be evenly occupied by the fleet of drones under consideration. To optimize the overall view, the drones must be evenly distributed angularly along the shape. The proposed method enables virtual planar shape description, decentralized bearing angle assignment, drone movement from takeoff positions to locations along the shape, and collision avoidance. Furthermore, the method allows for the number of drones to change during the mission. The method has been tested both in simulation, through emulation, and in outdoor experiments with real drones. The obtained results demonstrate that the method is applicable in real-world contexts.

## 1. Introduction

Many multi-robot systems, including swarm robotics, are inspired by collective behaviors observed in nature. The topic has emerged as a promising concept with numerous applications across various domains [1]. The development of key elements underlying self-organization, namely, collaboration, cooperation, and robustness, has enabled breakthroughs in many challenging and time-consuming tasks [2,3,4].

Multi-robot systems demonstrate their efficiency by sharing tasks among robots. For instance, they can rapidly cover a designated geographic area or perform search and tracking, particularly in the context of rescue missions [5,6,7]. Along the same lines, data exchange (including images and positions) can prevent collisions and assist the group in navigating complex environments, as is required for inspecting critical infrastructures [8].

Other advantages of these systems are their flexibility, adaptability, and robustness. Thanks to a decentralized approach facilitated by communication among group members, robots can reorganize themselves in response to dynamic and often unpredictable changes even when a few robots become inoperative. These properties prevent system failure, making multi-robot systems desirable for long-lasting tasks such as environmental monitoring, where individual robots may take over for others due to failure or lack of energy [9].

Behind these few examples, thousands of scientific papers and books have been published over the last few decades on the topic of swarm robotics (over 15,000 hits in google scholar for the last decade, and even more for multi-robot systems). These works range from purely theoretical studies to real experiments conducted in harsh environmental conditions. Researchers have explored centralized and decentralized methods as well as hybrid approaches, studied a wide array of robotic platforms, including aerial, ground, and underwater robots, and proposed applications spanning numerous domains. In addition, many surveys have been written in an attempt to classify the problems, approaches, methodologies, experimental platforms, and results.

The work presented in this paper focuses on multi-UAV systems, which are groups of identical Unmanned Aerial Vehicles (UAVs), sometimes referred as drones. While many works have been published in this field [10,11,12], the present paper specifically addresses the problem of pattern formation. In the context of this study, the goal consists of positioning a set of drones evenly along a predetermined planar shape, such as a circle, polygon, butterfly, etc. The term ‘evenly’ can be interpreted as maintaining equal distances between the drones or, alternatively, as arranging them at regular angular intervals from a fixed reference point along the shape. In this work, we investigate the latter option. Additionally, unlike several previous works described in [3], the shape itself is defined before the mission by a human operator. Thus, it does not result from interactions among the robots, as is usually the case for swarm robotics.

The original motivation for this work stemmed from an industrial accident that occurred in Normandy in 2019 (https://www.francetvinfo.fr/faits-divers/incendie/incendie-d-un-site-seveso-a-rouen/ (French television news), accessed on 29 November 2023). The Lubrizol petrochemical factory and several neighboring warehouses caught fire, and the toxic and dense cloud quickly spread high into the sky close to the city of Rouen. Due to its size and the proximity of houses, firefighters encountered great difficulty in controlling the fire. Later on, the debrief of this accident sparked discussions about possible future strategies for improving the conditions of action. One part of the discussion focused on the possibility of deploying a set of drones for observational purposes. The idea was to enable a multi-UAV system equipped with specific sensors [13] to surround the cloud at a relevant distance, with each drone pointing its camera towards the source of the fire. The current paper presents a decentralized and flexible solution for achieving this goal. The implementation of such a solution faces several problems.

Information should arrive continuously from the multi-UAV system; thus, due to battery capacity limitations, the composition of the group may change during the mission. This constraint eliminates solutions relying on a leader or any form of centralization, such as the one proposed by Raja and his colleagues [14]. Identifying the area of interest for observation and defining the shape delimiting this area is another issue. According to the situation, environment, and physical constraints, the area of interest may vary widely, invalidating all solutions targeting a specific set of shapes. In 2021, Huang and his colleagues [15] performed 3D representation with a UAV fleet using graph theory. For *N* UAVs, each 3D model is meshed by *N* nodes at specific coordinates. When an event transition is triggered, the fleet of UAVs moves from one shape to another by solving a task assignment optimization problem and planning a collision-free trajectory. An artificial potential field is used to manage obstacle avoidance in real-time. This work shows a concrete realization of a robust system with six UAVs; however, the model for each desired shape is meshed by a central machine beforehand, and the number of drones is fixed.

In order to define a wide variety of shapes delimiting potential areas of interest, we propose the use of Fourier Descriptors (FDs) [16,17,18]. When coupled with the Fourier Transform (FT), FDs allow a planar formation shape to be defined as a virtual rigid-body structure. Methods based on FDs ensure the representation and retrieval of a shape. They derive from a shape signature (in general, a 1D function) representing 2D areas or boundaries. Zhang and Lu [19] compared several methods based on FDs on a set of complex shapes. This approach provides results for the centroid distance with better robustness, convergence speed, and computation complexity, and is able to handle open curves. The FT approximates the shape signature with a finite number of harmonics in order to transform an unknown function into a low-computational sum of sinusoidal functions. To cope with the reactivity and flexibility constraints of decentralized networks, we decided to use a composition of virtual actions [20,21,22] to ensure collision avoidance. Each UAV adjusts its velocity according to the presence of static or dynamic obstacles in a defined range.

In the Section 2, the FD and FT formulation used to depict the desired formation is described. The decentralized allocation of the bearing angle between robots when the number of UAVs is known prior to the mission and when this number changes during the mission is described and explained in Section 3. Section 4 discusses modeling and control, along with obstacle avoidance management. The simulation protocols and experiments are presented in Section 5, followed by the results and commentary in Section 5.3.

## 2. Designing Planar Shapes with Fourier Descriptors

### 2.1. Problem Statement

It is desirable to have a way to control the formation regardless of the number of UAVs. The formation should be maintained when the number of UAVs varies over time. For certain applications, conditions such as the number of deployed UAVs, target movement, objectives, etc., may vary during the execution of the mission. These variations may entail a change in the formation, such as its size, position, orientation, etc. Thus, it is appropriate to seek a way of describing the pattern irrespective of its absolute position, orientation, and size.

One way to ensure this is to use a combination of Fourier Descriptors (FDs) and the Fourier Transform (FT). This method allows shapes to be described through modifications while maintaining formation coherence using three parameters: amplitude, phase, and reference point.

In this study, we chose to equally distribute the UAVs (index i) around the shape with bearing angles ($\beta_{i}$) computed according to the number of available UAVs. It is worth noting that the number of UAVs can be unknown a priori; the decentralized method enabling the determination and allocation of the bearing angles between UAVs is described in Section 3. Note that the term ‘equally’ (or ‘evenly’) refers to the angular distance from a UAV to a fixed reference point, not the Euclidean distance to that point or between UAVs. From now on, we consider that each UAV knows the bearing angle to be reached, which is implemented in the high-level control as a direction instruction.

Here, we want the UAV to find the coordinates of the point to be reached ($M_{i}$) while only knowing the reference point of the shape. Therefore, we have designed our functions to fit a real cyclic period of 2π by describing them in polar coordinates from this point. These functions do not need to be continuous; they can be sampled or piecewise continuous. To allow the planar shape to rotate in 3D, a rotational quaternion is provided to all UAVs alongside the harmonics from the FT.

**Figure 1:**
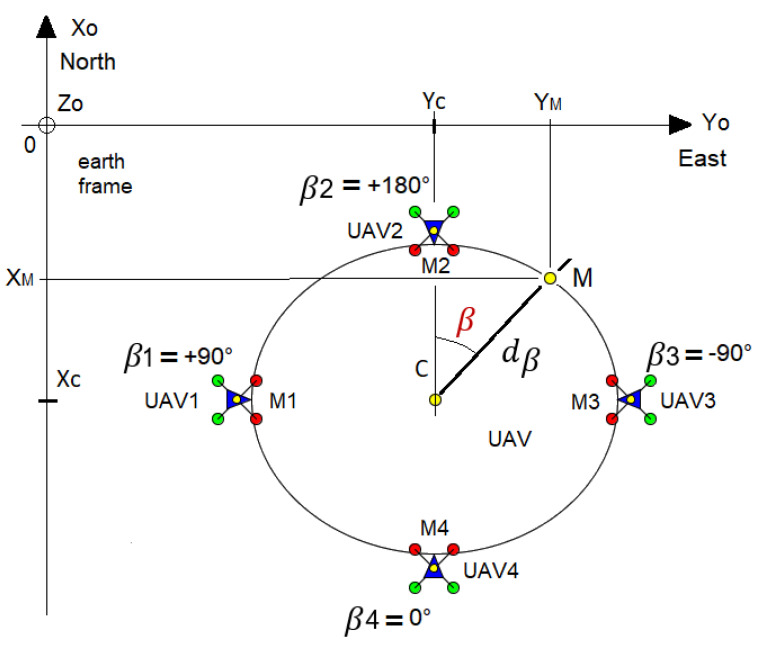
Example of an angular equidistribution of UAVs around an elliptical shape with its center at *C*. The red and green dots allow distinguishing the front from the back of the drone, with red indicating the front and green indicating the back.

Each UAV has to regulate their distance errors from the point towards zero in order to reach ($M_{i}$). At all times, they must point towards the reference point, which is the opposite of the direction $\beta_{i}$ (Figure 1).

### 2.2. Fourier Descriptor

**Figure 2:**
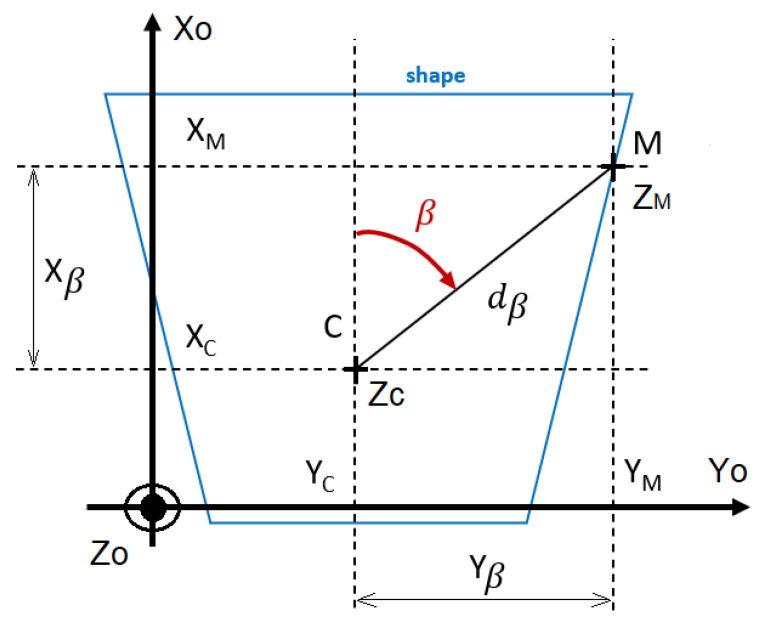
Example of a closed curve defined by $d_{\beta }$ with FD from the reference point *C*.

Fourier descriptors [16,17,19] describe a planar function $d_{\beta }$ with a single variable β from a reference point $M(X_{M},Y_{M})$ (Figure 2). This one-dimensional function $d_{\beta }$ is called the shape signature, and must be periodic.

In this study, we use the centroid distance to describe the signature shape $d_{\beta }$ of the desired curve (Figure 1 and Figure 2), as it captures and retrieves complex patterns with the most accuracy [19]. Additionally, it matches the polar description used to retrieve the desired position. It is expressed by the distance of the boundary points (*M*) from the centroid (*C*) of the shape. To match our problem statement, the reference point *C* can be any point as long as one angle provides one possible distance.

Discrete Fourier Transformation (DFT) is applied to retrieve the harmonics (1). Their number $f_{max}$ depends on the sampling frequency $f_{e}$ of the continuous function of the shape signature with respect the Nyquist–Shannon condition (2); *N* denotes the number of samples generated, and $\beta_{t}$ is the associated angle of the sample *t* parameter of the signature function.

(1) $$\begin{array}{c} a_{n}=\frac{1}{N}\sum_{t=1}^{N}d_{\beta_{t}}\exp\left(\frac{-j2\pi nt}{N}\right)\;\\ Re\left(a_{n}\right)=\frac{1}{N}\sum_{t=1}^{N}d_{\beta_{t}}\cos\left(\frac{2\pi nt}{N}\right)\;\\ Im\left(a_{n}\right)=\frac{1}{N}\sum_{t=1}^{N}d_{\beta_{t}}\sin\left(\frac{-2\pi nt}{N}\right) \end{array}$$

(2) $$f_{max}<\frac{f_{e}}{2}$$

The Fourier coefficients are determined using the DFT. The distance $d_{\beta }$ from the reference point is computed with the bearing angle β and the coefficients:

(3) $$\left\{\begin{array}{c} \phi_{n}=atan2\left(Im\left(a_{n}\right),Re\left(a_{n}\right)\right)\\ X_{\beta }=\sum_{n=0}^{N+1}\epsilon \left|a_{n}\right|\cos\left(n\beta +\phi_{n}\right)\\ Y_{\beta }=\sum_{n=0}^{N+1}\epsilon \left|a_{n}\right|\sin\left(n\beta +\phi_{n}\right)\\ d_{\beta }=\sqrt{X_{\beta }^{2}+Y_{\beta }^{2}} \end{array}\right.$$

$\phi_{n}$: phase of the *n*th harmonic

$d_{\beta }$: distance from the reference point *C* to the point *M*

β: angle between the *X* (north) axis and the direction defined by point *M* and the reference point *C*

$X_{\beta }$: distance along the *X* axis (north) of point *M* from the reference point *C*

$Y_{\beta }$: distance along the *Y* axis (east) of point *M* from the reference point *C*

### 2.3. Spatial Rotations

At this state, the position that is reached is determined by the altitude of the reference point $Z_{c}$, and is purely planar (Figure 2). Thus, a quaternion is defined to provide the third dimension of the planar shape, allowing 3D rotations. This only requires a revolutionary axis coupled with an angle.

Let *q* the initial quaternion resulting from DF and let FT be defined as follows:

(4) $$q=\left\{\begin{array}{c} a_{1}=1\\ v_{1}=\left\{\begin{array}{c} \cos\left(\beta \right)\\ \sin\left(\beta \right)\\ 0 \end{array}\right. \end{array}\right.$$

The rotational quaternion $q_{rot}$ is interpreted from the rotational angle $\alpha_{rot}$ and the normalized vector ${\left(x_{rot},y_{rot},z_{rot}\right)}^{T}$:

(5) $$q_{rot}=\left\{\begin{array}{c} a_{2}=\cos\left(\alpha_{rot}/2\right)\\ v_{2}=\left\{\begin{array}{c} \sin\left(\alpha_{rot}/2\right)*x_{rot}\\ \sin\left(\alpha_{rot}/2\right)*y_{rot}\\ \sin\left(\alpha_{rot}/2\right)*z_{rot} \end{array}\right. \end{array}\right.$$

The new normalized position vector $\underline{X}$, which is the result of the rotation of *q* around $q_{rot}$, can be expressed as

(6) $$\underline{X}=2*\left(v_{1}\cdot v_{2}\right)*v_{2}+a_{2}^{2}-\left(v_{2}\cdot v_{2}\right)*v_{1}+2*a_{2}*\left(v_{2}⊗v_{1}\right).$$

Next, the normalized position vector $\underline{X}$ is multiplied by the distance $d_{\beta }$ to retrieve the desired position of point *M*.

To conclude, the management of the formation involves leading each UAV *i* to their point $M_{i}$ defined around the 3D shape ($\beta ,d_{\beta }$) while avoiding obstacles and collisions.

In summary, the method allows each drone to obtain its destination position belonging to the shape using four main information: the reference point of the shape, the harmonics, magnetic north, and the bearing angle. In the context of this work, we additionally want this whole process to be decentralized in order to guarantee its flexibility and robustness. Flexibility means that before the mission any drone might be replaced by another one without the need to assign it a unique identifier. Robustness means that drones may leave the formation during the mission, while others can arrive and insert themselves into the shape. Regarding the necessary information for performing the mission, only one parameter has to be determined in a decentralized and online way for the dynamic version. Notably, the reference point and harmonics are predefined mission parameters that remain constant throughout the mission. As far as the UAVs are concerned, magnetic north is assumed to be common knowledge. Thus, the only information that needs to be computed is the bearing angle. Allocating the bearing angles to the drones prior to the mission as part of their parameters is a possibility; however, this choice would make the method less flexible and less robust. In such a case, the bearing angles would have to be individually transmitted to each drone by a central device, which implies a unique identifier for each drone, which contradicts the core principles of decentralized algorithms. Furthermore, such an approach reduces robustness, as the number of drones cannot change during the mission. The next section addresses these issues by presenting the algorithms used to ensure decentralization.

## 3. Decentralized Allocation of Bearing Angles

In this section, we present algorithms that enable drones to self-determine their bearing angles through message exchanges. These algorithms are executed asynchronously by each drone. In all cases, the drones do not have identifiers. Thus, all broadcast messages are anonymous. Three cases are investigated:

1. Static and reliable communication: the size of the group (number of drones) is known by every drone and no messages are lost during the exchanges.
2. Static and unreliable communication: the number of UAVs is known and messages may be lost during communication.
3. Dynamic and reliable communication: the number of drones is unknown and may change during the mission, meaning that drones may leave the formation and new drones may join it, while no message are lost during communication.

In all scenarios, the drones communicate by broadcasting messages and the communication topology is a fully connected graph. Thus, no routing is needed. All of the drones have a compass, enabling every drone to consider magnetic north as the reference null angle. The drones have to be equally distributed over the shape; thus, computing an angle is equivalent to each of them choosing a number between 0 and N−1, where *N* refers to the number of drones. These numbers are called *positions*, and two drones cannot have the same position. Note that for the 3 (dynamic-reliable-com) case *N* can change during the mission. In this study, the term *consensus* refers to the situation where each position has been chosen by one and only one drone. The algorithms presented in the sequelae are designed such that consensus is reached through a decentralized process.

### 3.1. Known Constant Number of UAVs

For this first case, we assume that several conditions are fulfilled. We suppose that the drones communicate by broadcasting messages and that each broadcast message is received by the other drones. We suppose that the number of drones initially equals *N* and that no drone leaves or joins the group during the mission.

The principle lies in the use of an array of Boolean values. In this array, cell *i* is *true* when position *i* has been chosen by a drone and is *false* when no drone has chosen this value. When necessary, drones broadcast both their array of Booleans and their chosen position. If two drones have chosen the same position, there is a conflict. Thus, upon reception of such a message, the drone compares its position and the received one; in the case of conflict, it performs a new random choice of position such that its corresponding cell in the array contains *false*. The method is formally written out in Algorithm A1 (and see Appendix A.2).

To measure the performance of the algorithm, the asynchronous execution of the algorithm by each drone was obtained through a multi-threaded simulation. Each drone was assigned to an independent thread that executed the algorithm. Two scenarios were considered. In the first scenario, message loss was not considered, while in the second scenario a substantial percentage of broadcast messages were lost.

**Figure 3:**
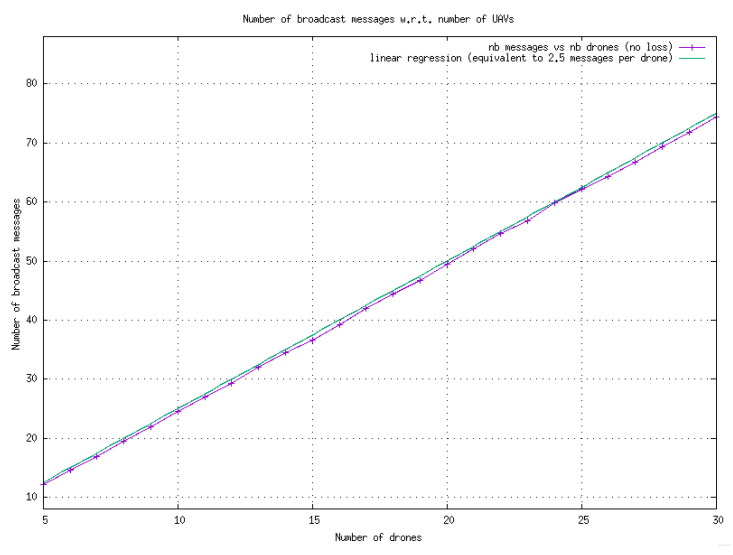
Average total number of messages broadcast by drones for reaching consensus during the bearing angle choice phase. On average, 2.5 messages are broadcast by the drones. Each point on the graph corresponds to 100 runs. In this scenario, it is assumed that no messages are lost.

The algorithm is highly efficient in the scenario without message loss, with an average of only 2.5 broadcast messages per drone required to achieve consensus. In this process, each drone selects a unique number from [0,N−1] such that each number is associated with one and only one drone. The experimental results are reported in Figure 3.

In the second scenario, broadcast messages loss may lead to a deadlock. In case of a conflict, the two involved drones have to choose another number and broadcast their new choice. If one of the two messages is lost, the other members of the group receive one message from the other drone with a cell containing “false”; as the other message is never received, the drones may wait forever. To cope with this issue, a timeout mechanism is introduced into the algorithm. If a drone does not receive any message after a given period of time and continues to have cells containing “false” within its array, it repeats broadcasting of its position array and its own position. This new broadcast triggers allows the correct positions to be marked “false” in the received array. Thus, the condition in line 5 of Algorithm A1 becomes: **if** change OR timeout **then**.

**Figure 4:**
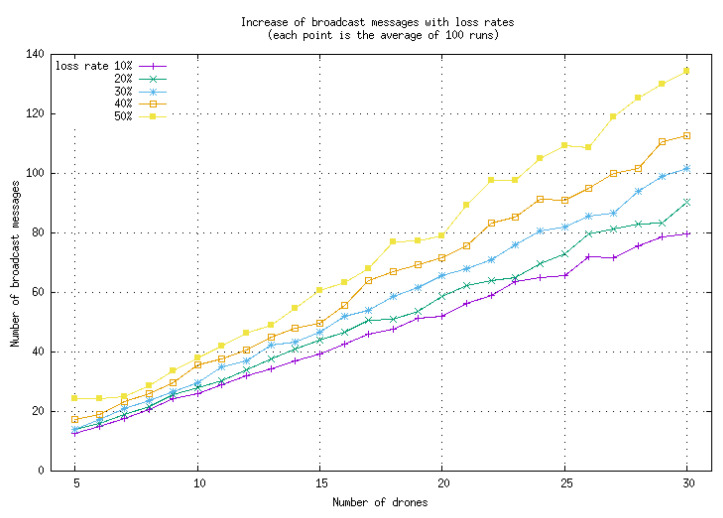
Average number of broadcast messages needed when a given percentage of messages are lost. The considered loss rates range from 10 to 50%. The increase in the number of broadcast messages remains limited even when the rate of loss is high.

As reported in Figure 4, the number of broadcast messages increases with the percentage of lost messages. However, the method remains very robust, as large loss percentages (up to 50%) do not prevent the algorithm from reaching consensus. Furthermore, the number of additional messages needed to cope with the lost messages grows linearly with the number of drones (with a factor close to 4.5 for a 50% loss rate), making communications congestion unlikely.

### 3.2. Dynamically Changing Number of UAVs

For this last scenario, the composition of the group is dynamic. Two opposite events may occur: a drone joins the group or a drone leaves the group. We assume several conditions to be true:

- Communication is assumed to be reliable, i.e., no messages are lost.
- There is enough time between two events (join or leave) for the group to reach a consensus on each drone’s respective chosen position.
- The topology of the communication network is a fully connected graph; thus, no routing is needed.
- It is assumed that messages are received in the same chronological order in which they are emitted. Specifically, it is expected that a message sent at time *t* by drone $D_{i}$ is received by all other drones before any message broadcast at a later time $t^{\prime }>t$ by drone $D_{j}$. This assumption can be considered generally valid in the context of a fully connected communication topology.
- The drones process their messages in First-In First-Out (FIFO) order.

Because of these changes, unlike the previous scenario the allocation of bearing angles is a never-ending process. The method lies in the broadcast messages. Each message contains three fields: the position chosen by the drone, its array of positions (Boolean values), and the type of the message.
My position[true,true,false,⋯,true]MSG_TYPE

Three types of messages are considered:

- JOIN: when a drone wants to join the group, it broadcasts a JOIN-type message with its position (0 by default) and an array of only one cell containing *true*.
- LEAVE: when a drone leaves the group, it broadcasts a LEAVE-type message with its current position and the corresponding array with *false* at its own position.
- UPDATE: several situations may lead to the emitting of an UPDATE-type message:
  1. When a drone changes its position (caused by a conflict), it broadcasts a new UPDATE-type message with its newly chosen position and the new array with the Boolean *false* in the cell of its former position.
  2. When a drone receives a JOIN-type or LEAVE-type message, it modifies its array of positions according to the situation and broadcasts an UPDATE-type message.
  3. For newly arriving drones, a drone updates the size of its array after receiving an UPDATE-type message.

As an example, consider a drone *D* that has chosen a position *j*. Its array of positions contains *true* at index *i* (≠j) if and only if it receives a message from another drone claiming *i* as its own position. If *D* receives a message with *false* at index *j* in the array, it broadcasts a new UPDATE message claiming *j* as its position with *true* at index *j* in the array.

After a first consensus has been found (i.e., the drones have all chosen their respective positions), the decentralized method positions any newly arriving drone at the last position. When a drone leaves the group, only those drones with a larger position value change it, decreasing its value by 1. This ensures minimal distance changes for all drones in both cases and reduces the likelihood of collisions.

The algorithm is composed of two processes: a reception process that gathers received messages into a FIFO structure (mailbox in the Algorithms) and the main process described by Algorithms A2 and A3 (provided in Appendix A).

**Figure 5:**
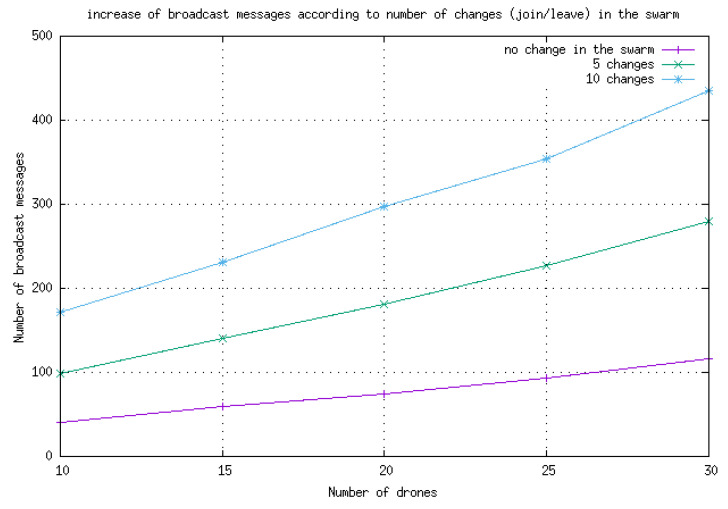
Average number of broadcast messages when drones join or drones leave the group; the considered numbers of changes are 0, 5, and 10.

**Figure 6:**
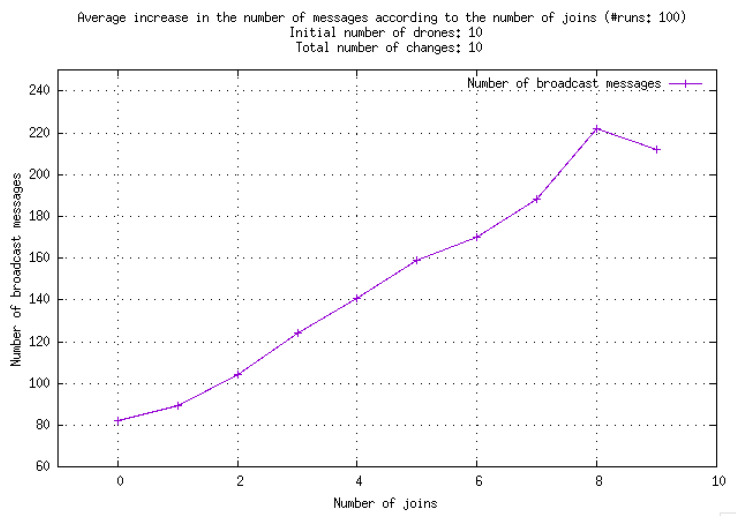
Increase in the number of broadcast messages with respect to the number of drones joining the group. The considered number of changes is always 10; thus, three joins means that three drones joined the group and seven left it.

In Figure 5, it can be observed that the number of broadcast messages increases linearly with the number of changes occurring within the group. However, this increase is mainly due to drones joining the group, as illustrated by Figure 6.

## 4. Control Framework

**Figure 7:**
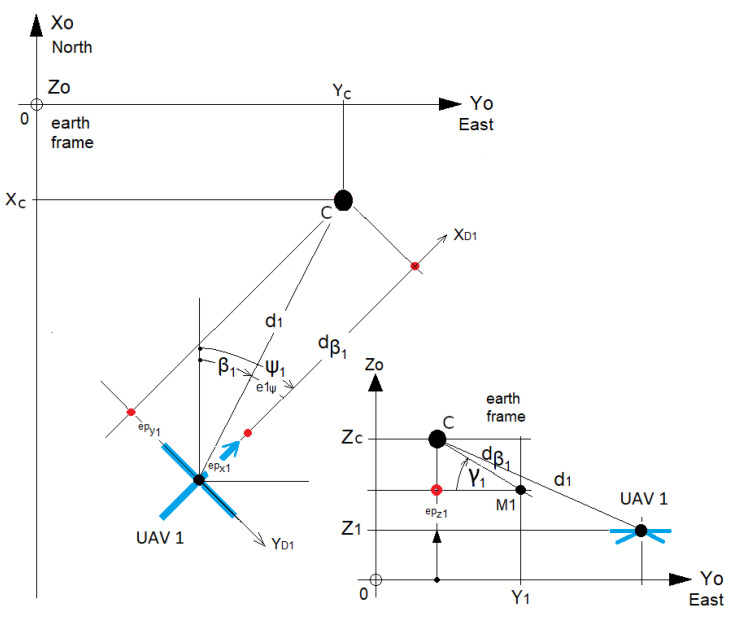
Definition of the position errors for UAV 1.

The formation of UAVs (marked with an index *i*) must surround a predefined shape computed using Fourier descriptors. The UAVs orient themselves towards the desired direction $\beta_{i}$ (Figure 1), defined as the angle between the *X* axis and the direction of the UAV in the North/East/Down (NED) reference. The UAVs must reach their target position $M_{i}$ while avoiding each other to prevent collisions. The GPS coordinates of the reference point (*C*) are known. The Fourier descriptor returns the distance $d_{\beta_{i}}$ and altitude angle $\gamma_{i}$ from point *C* for a given angle $\beta_{i}$ and a rotational quaternion (see Section 2, Figure 7). Each UAV has to regulate their distance errors with respect to the point to be reached ($M_{i}$) toward zero while pointing towards it according to the desired direction $\beta_{i}$ (Figure 1). Double exponential smoothing [23,24,25] is performed in real time to compute a filtered estimation and predict the distance $\hat{d_{i}}$ and bearing angle $\hat{\psi_{i}}$.

### 4.1. Control Law

The main goal of the proposed cascade controller is to regulate the position of the UAV compared to the fixed target point while maintaining good safety conditions. For this, two different controllers have been designed and implemented. The speed controller (inner loop) takes into account the speed errors according to the x, y, and z axes and their respective variations as functions of time. To ensure safe behavior, a first saturation function (f) is implemented to limit the pitch/yaw angles and the vertical acceleration when the sum of the speed errors (weighted by three parameters that have to be tuned) exceeds a given speed. Below this limit, the UAV starts to regulate its speed; otherwise, it moves at constant pitch/yaw angles and vertical acceleration. The speed controller outputs are the reference inputs (attitude and vertical acceleration) of the flight controller of the UAV. The three parameters of the speed controller have to be set first in order to obtain a closed loop behavior for the the speed control (inner loop) that is significantly faster than the position control (outer loop). The position controller (outer loop) takes into account the position errors according to the x, y, and z axes and their variations as functions of time. To ensure safe behavior, a second saturation function (f) is implemented to limit the speed according to x, y, and z when the sum of the position errors (weighted by three coefficients that have to be tuned) exceeds a given distance. Below this limit, the UAV starts to regulate its position compared to the target point; otherwise, it moves at a maximum constant speed. The outputs of the position controller are the reference inputs of the speed controller.

The attitude control was designed by Squadrone Systems [26]; the control inputs of the UAVs, namely, the roll (Φ), pitch (Θ), yaw (Ψ), and vertical acceleration ($A_{Z}$), are available in the C++ API. Below, we describe the control law for $UAV_{1}$.

#### 4.1.1. Velocity Regulation

The filtered velocity errors ${\hat{\epsilon v}}_{1}$ of $UAV_{1}$ are as follows (Figure 7):

(7) $${\dot{\epsilon v}}_{1}:\left\{\begin{array}{c} {\dot{ev}}_{x1}=V_{x1}-{\hat{V}}_{x1}\\ {\dot{ev}}_{y1}=V_{y1}-{\hat{V}}_{y1}\\ {\dot{ev}}_{z1}=V_{z1}-{\hat{V}}_{z1} \end{array}\right.$$

For $UAV_{1}$, the velocity control objective is as follows (8):

(8) $$\lim_{t\to \infty }{\dot{\epsilon v}}_{1}\left(t\right)=0:\left\{\begin{array}{c} \lim_{t\to \infty }{\dot{ev}}_{x1}\left(t\right)=0\\ \lim_{t\to \infty }{\dot{ev}}_{y1}\left(t\right)=0\\ \lim_{t\to \infty }{\dot{ev}}_{z1}\left(t\right)=0 \end{array}\right.$$

To ensure the control objective (8), the following velocity control law (9) has been implemented:

(9) $$\begin{array}{cc} \; & \left[\begin{array}{c} {\dot{\hat{ev}}}_{x1}\\ {\dot{\hat{ev}}}_{y1}\\ {\dot{\hat{ev}}}_{z1} \end{array}\right]=-\left[\begin{array}{c} f(\sum_{j=1}^{3}kv_{xj}.\epsilon v_{xj},\bar{\Theta_{1}},{\bar{Vx}}_{1},0)\\ f(\sum_{j=1}^{3}kv_{yj}.\epsilon v_{yj},\bar{\Phi_{1}},{\bar{Vy}}_{1},0)\\ f(\sum_{j=1}^{3}kv_{zj}.\epsilon v_{zj},{\bar{Az}}_{1},{\bar{Vz}}_{1},g) \end{array}\right]=-{\vec{V}}_{CMD1} \end{array}$$

(10) $${\vec{V}}_{CMD1}=\left[\begin{array}{c} f(\sum_{j=1}^{3}kv_{xj}.\epsilon v_{xj},\bar{\Theta_{1}},{\bar{Vx}}_{1},0)\\ f(\sum_{j=1}^{3}kv_{yj}.\epsilon v_{yj},\bar{\Phi_{1}},{\bar{Vy}}_{1},0)\\ f(\sum_{j=1}^{3}kv_{zj}.\epsilon v_{zj},\bar{Az_{1}},{\bar{Vz}}_{1},g) \end{array}\right]$$

$$\begin{array}{cccccc} \epsilon v_{x1}={\hat{ev}}_{x1} & , & \epsilon v_{x2}={\dot{\hat{ev}}}_{x1} & , & {\dot{\epsilon v}}_{x3}={\hat{ev}}_{x1} & ;\\ \epsilon v_{y1}={\hat{ev}}_{y1} & , & \epsilon v_{y2}={\dot{\hat{ev}}}_{y1} & , & {\dot{\epsilon v}}_{y3}={\hat{ev}}_{y1} & ;\\ \epsilon v_{z1}={\hat{ev}}_{z1} & , & \epsilon v_{z2}={\dot{\hat{ev}}}_{z1} & , & {\dot{\epsilon v}}_{z3}={\hat{ev}}_{z1} & ; \end{array}$$

$kv_{xj}$, $kv_{yj}$, $kv_{zj}$: the coefficients to be customized,

${\vec{V}}_{CMD1}$ = ($\Theta_{1}$, $\Phi_{1}$, $Az_{1}$)${}^{T}$: the command vector of $UAV_{1}$,

${\bar{\Theta }}_{1}$, ${\bar{\Phi }}_{1}$, ${\bar{Az}}_{1}$: the maximal angular positions and vertical acceleration, respectively, reached when the velocity errors are higher or equal to the velocities ${\bar{Vx}}_{1}$, ${\bar{Vy}}_{1}$, ${\bar{Vz}}_{1}$,

*g* is the gravity acceleration in m/s${}^{2}$.

Note that the full command vector of $UAV_{1}$ is ${\vec{V}}_{CMD1}$ = ($\Theta_{1}$, $\Phi_{1}$, $\Psi_{1}$, $Az_{1}$)${}^{T}$

#### 4.1.2. Position Regulation

To reach the desired orientation $\beta_{1}$ provided by the Fourier descriptors, we only have to obtain $\beta_{1}$ as the reference value $\Psi_{1}$ of the yaw control:

$$\Psi_{1}=\beta_{1}$$

The filtered position errors ${\hat{\epsilon p}}_{1}$ of $UAV_{1}$ are as follows (Figure 7):

(11) $${\hat{\epsilon p}}_{1}:\left\{\begin{array}{c} {\hat{ep}}_{x1}={\hat{d}}_{1}\cos\left(\beta_{1}-{\hat{\psi }}_{1}\right)\cos\left(\gamma_{1}\right)-d_{\beta_{1}}\\ {\hat{ep}}_{y1}={\hat{d}}_{1}\sin\left(\beta_{1}-{\hat{\psi }}_{1}\right)\sin\left(\gamma_{1}\right)\\ {\hat{ep}}_{z1}=\left(Z_{c}-d_{\beta_{1}}\sin\left(\gamma_{1}\right)\right)-Z_{1} \end{array}\right.$$

$\beta_{1}$: the bearing angle of $UAV_{1}$ computed by the Fourier descriptors (Figure 2),

$d_{\beta_{1}}$: the desired distance of $UAV_{1}$ computed by the Fourier descriptors (Figure 2),

$\gamma_{1}$: the altitude angle between the target point M1 and reference point *C* computed by the Fourier descriptors and the quaternion (Figure 2),

${\hat{d}}_{1}$: the filtered distance between $UAV_{1}$ and the reference point *C*,

${\hat{\psi }}_{1}$: the filtered bearing angle (yaw) of $UAV_{1}$,

$Z_{C}$, $Z_{1}$: the respective altitudes of the reference point *C* and $UAV_{1}$.

For $UAV_{1}$, the position control objective is as follows (12):

(12) $$\lim_{t\to \infty }{\hat{\epsilon p}}_{1}\left(t\right)=0:\left\{\begin{array}{c} \lim_{t\to \infty }{\hat{ep}}_{x1}\left(t\right)=0\\ \lim_{t\to \infty }{\hat{ep}}_{y1}\left(t\right)=0\\ \lim_{t\to \infty }{\hat{ep}}_{z1}\left(t\right)=0 \end{array}\right.$$

To ensure the control objective (12), the following position control law (13) has been implemented:

(13) $$\begin{array}{cc} \; & \left[\begin{array}{c} {\dot{\hat{ep}}}_{x1}\\ {\dot{\hat{ep}}}_{y1}\\ {\dot{\hat{ep}}}_{z1} \end{array}\right]=-\left[\begin{array}{c} f(\sum_{j=1}^{3}kp_{xj}.\epsilon p_{xj},{\bar{Vx}}_{1},{\bar{Dx}}_{1},0)\\ f(\sum_{j=1}^{3}kp_{yj}.\epsilon p_{yj},{\bar{Vy}}_{1},{\bar{Dy}}_{1},0)\\ f(\sum_{j=1}^{3}kp_{zj}.\epsilon p_{zj},{\bar{Vz}}_{1},{\bar{Dz}}_{1},0) \end{array}\right]=-\vec{V_{1}} \end{array}$$

We obtain

(14) $$⟹\vec{V_{1}}=\left[\begin{array}{c} f(\sum_{j=1}^{3}kp_{xj}.\epsilon p_{xj},{\bar{Vx}}_{1},{\bar{Dx}}_{1},0)\\ f(\sum_{j=1}^{3}kp_{yj}.\epsilon p_{yj},{\bar{Vy}}_{1},{\bar{Dy}}_{1},0)\\ f(\sum_{j=1}^{3}kp_{zj}.\epsilon p_{zj},{\bar{Vz}}_{1},{\bar{Dz}}_{1},0) \end{array}\right]$$

$$\begin{array}{cccccc} \epsilon p_{x1}={\hat{ep}}_{x1} & , & \epsilon p_{x2}={\dot{\hat{ep}}}_{x1} & , & \dot{\epsilon p_{x3}}=\hat{ep_{x1}} & ,\\ \epsilon p_{y1}={\hat{ep}}_{y1} & , & \epsilon p_{y2}={\dot{\hat{ep}}}_{y1} & , & \dot{\epsilon p_{y3}}={\hat{ep}}_{y1} & ;\\ \epsilon p_{z1}={\hat{ep}}_{z1} & , & \epsilon p_{z2}={\dot{\hat{ep}}}_{z1} & , & \dot{\epsilon p_{z3}}={\hat{ep}}_{z1} & ; \end{array}$$

$kp_{xj}$, $kp_{yj}$, $kp_{zj}$: the coefficients to be customized,

$\vec{V_{1}}=[Vx_{1}$, $Vy_{1}$, $Vz_{1}{]}^{T}$: the linear velocity vector of $UAV_{1}$,

${\bar{Vx}}_{1}$, ${\bar{Vy}}_{1}$, ${\bar{Vz}}_{1}$: the maximal linear velocities reached when the position errors are respectively higher or equal to the distance ${\bar{Dx}}_{1}$, ${\bar{Dy}}_{1}$, ${\bar{Dz}}_{1}$.

The saturation function *f* is defined as follows:

(15) $$f\left(\epsilon ,\;\max,\;\lim,\;\mathrm{offset}\right)=\begin{array}{ccc} -\max+\;\mathrm{offset} & \mathrm{if} & \epsilon <-\lim\\ \frac{\max}{\lim}\epsilon +\;\mathrm{offset} & \mathrm{if} & -\lim\leq \epsilon \leq \lim\\ \max+\;\mathrm{offset} & \mathrm{if} & \epsilon >\lim \end{array}$$

Note that the manufacturer [26] provided us with the dynamic closed loop model of the UAV, which corresponds to the attitude and vertical acceleration control. We used this dynamic model in Matlab/Simulink to first tune the three parameters of the speed controller and then the three parameters of the position controller in order to obtain the shortest response time for each loop (pole placement) without static errors, damping, or oscillations.

#### 4.1.3. Stability Analysis

For the speed controller (inner loop), we defined three positive gains corresponding to the slopes (max/lim) of the saturation function in the intervals [$-\bar{Vx_{1}}$…+$\bar{Vx_{1}}$], [$-\bar{Vy_{1}}$…+$\bar{Vy_{1}}$], and [$-\bar{Vz_{1}}$…+$\bar{Vz_{1}}$]:

$$\lambda_{x1}=\bar{\Theta_{1}}/\bar{Vx_{1}},\lambda_{y1}=\bar{\Phi_{1}}/\bar{Vy_{1}},\lambda_{z1}=\bar{Az_{1}}/\bar{Vz_{1}}$$

Consider the following (positive) candidate Lyapunov function:

$$Vs_{1}=\left({\hat{\epsilon v_{x1}}}^{2}+{\hat{\epsilon v_{y1}}}^{2}+{\hat{\epsilon v_{z1}}}^{2}\right)/2.$$

Its time derivative can be written as

$$\dot{Vs_{1}}=\left(\hat{\epsilon v_{x1}}\cdot \dot{\hat{\epsilon v_{x1}}}+\hat{\epsilon v_{y1}}\cdot \dot{\hat{\epsilon v_{y1}}}+\hat{\epsilon v_{z1}}\cdot \dot{\hat{\epsilon v_{z1}}}\right),$$

$$\dot{Vs_{1}}=\hat{\epsilon v_{x1}}\cdot \left(-\lambda_{x1}\cdot \hat{\epsilon v_{x1}}\right)+\hat{\epsilon v_{y1}}\cdot \left(-\lambda_{y1}\cdot \hat{\epsilon v_{y1}}\right)+\hat{\epsilon v_{z1}}\cdot \left(-\lambda_{z1}\cdot \hat{\epsilon v_{z1}}\right).$$

In conclusion, it is apparent that $\dot{Vs_{1}}<0$, as $\lambda_{x1}$, $\lambda_{y1}$, and $\lambda_{z1}$ are all positive gains.

For the position controller (outer loop), we define three positive gains corresponding to the slopes (max/lim) of the saturation function in the intervals [$-\bar{Dx}$…+$\bar{Dx}$], [$-\bar{Dy}$…+$\bar{Dy}$], and [$-\bar{Dz}$…+$\bar{Dz}$]:

$$\gamma_{x1}=\bar{Vx_{1}}/\bar{Dx_{1}},\gamma_{y1}=\bar{Vy_{1}}/\bar{Dy_{1}},\gamma_{z1}=\bar{Vz_{1}}/\bar{Dz_{1}}$$

Let us consider the following (positive) candidate Lyapunov function:

$$Vp_{1}=\left({\hat{\epsilon p_{x1}}}^{2}+{\hat{\epsilon p_{y1}}}^{2}+{\hat{\epsilon p_{z1}}}^{2}\right)/2.$$

Its time derivative can be written as

$$\dot{Vp_{1}}=\left(\hat{\epsilon p_{x1}}\cdot \dot{\hat{\epsilon p_{x1}}}+\hat{\epsilon p_{y1}}\cdot \dot{\hat{\epsilon p_{y1}}}+\hat{\epsilon p_{z1}}\cdot \dot{\hat{\epsilon p_{z1}}}\right),$$

$$\dot{Vp_{1}}=\hat{\epsilon p_{x1}}\cdot \left(-\gamma_{x1}\cdot \hat{\epsilon p_{x1}}\right)+\hat{\epsilon p_{y1}}\cdot \left(-\gamma_{y1}\cdot \hat{\epsilon p_{y1}}\right)+\hat{\epsilon p_{z1}}\cdot \left(-\gamma_{z1}\cdot \hat{\epsilon p_{z1}}\right).$$

In conclusion, it is apparent that $\dot{Vp_{1}}<0$, as $\gamma_{x1}$, $\gamma_{y1}$, and $\gamma_{z1}$ are all positive gains.

#### 4.1.4. Collision Avoidance

**Figure 8:**
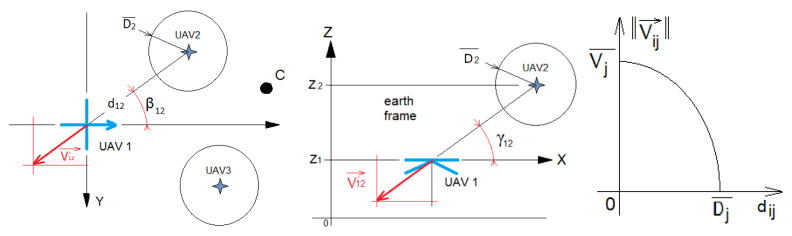
Collision avoidance scheme based on attractive and repulsive speeds, as described by Formulas (16) and (18).

The proposed collision avoidance method can be used with either static obstacles (poles, etc.,) or dynamic obstacles (other UAVs) if their GPS coordinates are known or sent in real time. Let $V_{i}$, the velocity of $UAV_{i}$, be the subtraction of a repulsive speed $V_{i}^{r}$ from an attractive one $V_{i}^{a}$ (10); $V_{i}^{r}$ is the repulsive effect applied by the neighboring $UAV_{j}$ on $UAV_{i}$. When $UAV_{i}$ crosses the defined protected circular area of $UAV_{j}$ (i.e., $D_{ij}$ is below a threshold ${\bar{D}}_{j}$), $UAV_{i}$ is subject to a repulsive effect homogeneous to a velocity with an intensity $|{\vec{V}}_{ij}|$ that changes according to the inter-UAV distance $d_{ij}$ (Figure 8). Each UAV computes its distance $D_{ij}$, altitude angle $\gamma_{ij}$, and bearing angle $\beta_{ij}$ with respect to its neighbor *j* using the Haversine function based on its GPS coordinates emitted in real time over the communications network. When including the collision avoidance method, Equation (14) becomes

(16) $$\begin{array}{cc} \; & \vec{V_{i}}=\left[\begin{array}{c} f\left(\sum_{j=1}^{3}kp_{xj}.\epsilon p_{xj},{\bar{Vx}}_{1},{\bar{Dx}}_{1},0\right)-\sum_{j=1,j\neq i}^{n}∥\vec{V_{ij}}∥\cos\left(\beta_{ij}-\psi_{i}\right)\cos\gamma_{ij}\\ f\left(\sum_{j=1}^{3}kp_{yj}.\epsilon p_{yj},{\bar{Vy}}_{1},{\bar{Dy}}_{1},0\right)-\sum_{j=1,j\neq i}^{n}∥\vec{V_{ij}}∥\sin\left(\beta_{ij}-\psi_{i}\right)\cos\gamma_{ij}\\ f\left(\sum_{j=1}^{3}kp_{zj}.\epsilon p_{zj},{\bar{Vz}}_{1},{\bar{Dz}}_{1},0\right)-\sum_{j=1,j\neq i}^{n}∥\vec{V_{ij}}∥\sin\gamma_{ij} \end{array}\right] \end{array}$$

with

(17) $$\begin{array}{cc} \; & \gamma_{ij}=\arctan\left(Z_{j}-Z_{i},d_{ij}\right)\\ \; & D_{ij}=\sqrt{d_{ij}^{2}+{\left(Z_{j}-Z_{i}\right)}^{2}} \end{array}$$

The intensity of the repulsive effect (Figure 8) applies when

(18) $$∥{\vec{V}}_{ij}∥=\left\{\begin{array}{ccc} {\bar{V}}_{j}\cos\left(\frac{\pi D_{ij}}{2{\bar{D}}_{j}}\right) & \mathrm{if} & D_{ij}<{\bar{D}}_{j}\\ 0 & \mathrm{else} \end{array}\right.$$

## 5. Simulations and Experiments

To demonstrate the decentralized coordination efficiency using FD and FT, several simulations were conducted on different shapes.

### 5.1. Simulations Description

**Figure 9:**
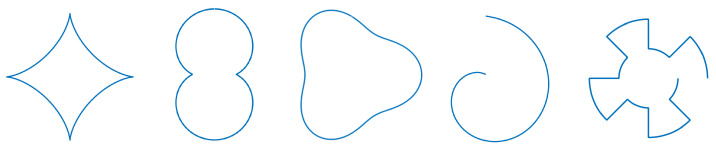
From left to right: the astroidal, peanut, pear, shell, and square signal shapes.

In keeping with the previous explanations, the shapes were restricted to be polar, continuous, and 2π periodical. This allows drones to compute their desired position from only the reference point and each drone’s desired bearing angle. Thus, we selected astroidal (star), peanut, pear, shell, and square signal shapes (see Equation (19) and Figure 9). This set was used to test the method’s robustness on asymmetrical, discontinuous, convex, and concave shapes.

(19) $$\begin{array}{c} \mathrm{astroidal}:\;f\left(x\right)={\left(|cos\left(x\right)\right|}^{n}+{\left|sin\left(x\right)\right|}^{n}{)}^{-\frac{1}{n}}\;;\;\forall x\in ]-\frac{\pi }{2};\frac{\pi }{2}[\;\mathrm{with}\;n=\frac{2}{3}\;\\ \mathrm{peanut}:\;f\left(x\right)=n+|cos\left(x\right)|\;;\;\forall x\in [0;2\pi [\;\mathrm{with}\;n=\frac{1}{2}\;\\ \mathrm{shell}:\;f\left(x\right)=x+2\;;\;\forall x\in [0;2\pi [\;\\ \mathrm{geoidal}/\mathrm{pear}:\;f\left(x\right)=5+\frac{\cos\left(3x\right)}{6}\;;\;\forall x\in [0;2\pi [\;\\ \mathrm{square}\;\mathrm{signal}:\;\left\{\begin{array}{c} f\left(x\right)=1\;;\;\forall x\in \left\{[0;\frac{\pi }{4}[,[\frac{\pi }{2};\frac{3\pi }{4}[,[\pi ;\frac{5\pi }{4}[,[\frac{3\pi }{2};\frac{7\pi }{4}[\right\}\;\\ f\left(x\right)=2\;;\;\forall x\in \left\{[\frac{\pi }{4};\frac{\pi }{2}[,[\frac{3\pi }{4};\pi [,[\frac{5\pi }{4};\frac{3\pi }{2}[,[\frac{7\pi }{4};2\pi [\right\} \end{array}\right. \end{array}$$

Each signature shape was sampled from 0 to 2π by 1000 points, allowing up to 500 harmonics without folding effects (i.e., the Nyquist–Shannon criteria). For all simulations, we used 250 harmonics to retrieve the described shape. For each simulation, the parameters were set to a scale factor of 20, no rotation, and (0,0) as the reference point. This was the setup used by default unless otherwise specified.

First, we evaluated our method for retrieving shapes to demonstrate its accuracy on the set of shapes. On the astroidal and the peanut formations, the *n* function parameter was varied to make the initial description more or less concave and convex, respectively.

In addition, these simulations were used to illustrate the impact of the number of harmonics used during the shape retrieval.

Transformations were applied to validate the non-variation of shape design in 3D space through modifications.

In the simulations, starting from their origin, the UAVs were required to equally distribute themselves angularly along the shape’s border while facing the reference point. Then, to depict the total shape and ensure shape continuity, we carried out runs in which one drone needed to follow the shape border by continuously incrementing its desired bearing angle ($+0.5^{\circ }$ per second).

Finally, a drone was deployed to reproduce the pear shape. A second drone was used to validate collision avoidance with both virtual and real obstacles.

### 5.2. Flight Simulations

**Figure 10:**
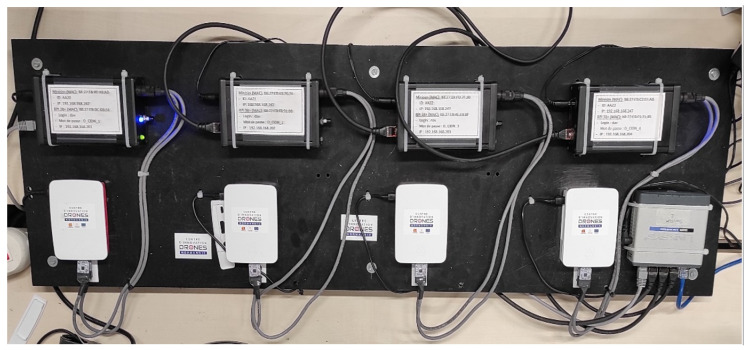
Squadrone Systems “MiniSim” simulators and their Raspberry PI 3B+ companion computers.

**Figure 11:**
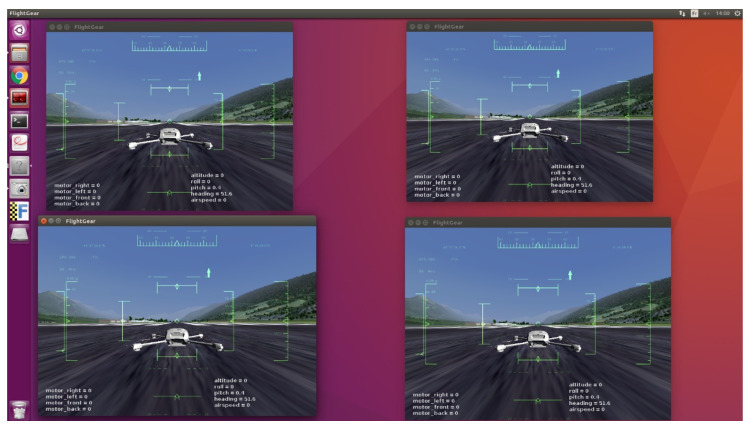
Flight visualisation on FlightGear during the emulation phase [28].

The first flight simulations were conducted on Processing [27] to simulate any number of drones. The second set of runs were conducted on a system composed of the following:

1. Four “MiniSim” simulators (designed by Squadrone Systems [26]) of the “hardware in the loop” (HITL) type, reproducing the dynamic behaviour of the UAVs (Figure 10);
2. Four embedded RaspBerry PI 3B+ companion computers, on which our algorithms were implemented in C++. These were the same embedded computers installed on the real UAVs.
3. An operator computer to connect the four embedded computers together for programming and executing the algorithms.
4. A flight simulator on a separate computer on the “MiniSim” simulators linked via rooter. Open-source FlightGear (v. 2020.3) [28] software was used to visualize the flight of the drones. This setup allowed several sessions to be run simultaneously in order to observe the flight of the group (one UAV per window, as illustrated on Figure 11).

This whole system emulates the workings and behavior of real drones in a field test without being subject to communication hazards.

### 5.3. Simulations Results

**Figure 12:**
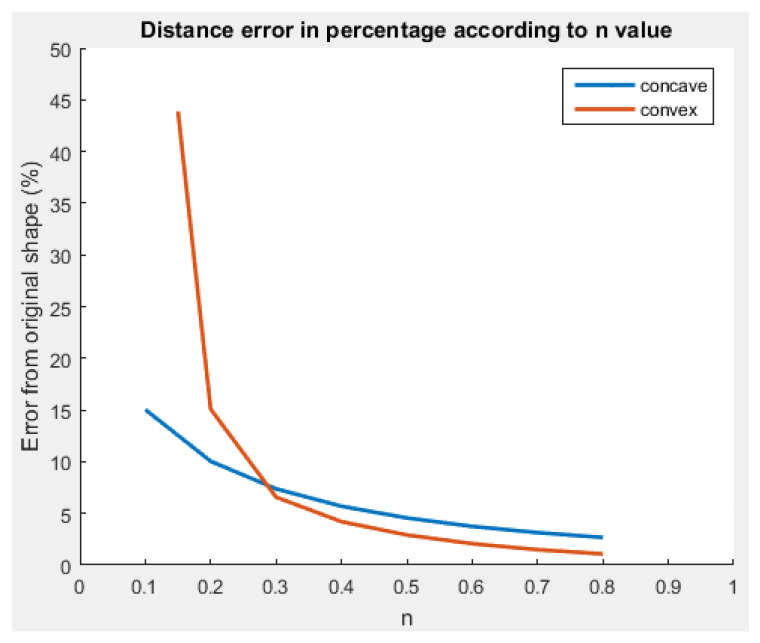
Evolution of the retrieval error according to the curve factor of the peanut and star shapes based on Table A1 and Table A2 in Appendix A.

We used Matlab 2015B [29] to evaluate our shape retrieval method on the set of formations presented in Figure 9. On the concave (peanut) and convex (star) shapes, we modified the curve factor *n* in Equation (19) to increase the given shape test panel. As a result, the more curvy the shape in Figure 12, the more the retrieval accuracy decreases. When the star shape is almost a straight line in each direction, the method transcription falls drastically to an error of almost 50%.

**Figure A3:**
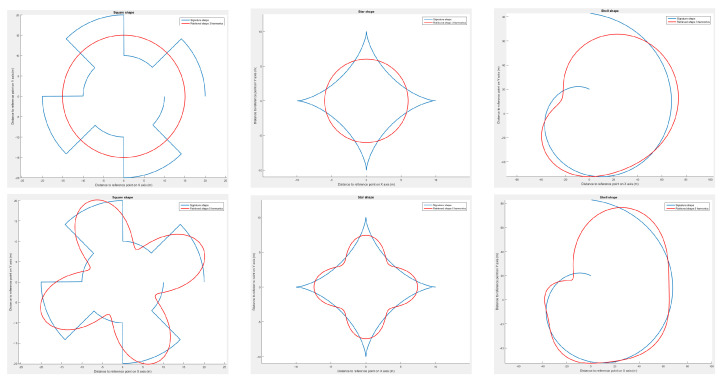

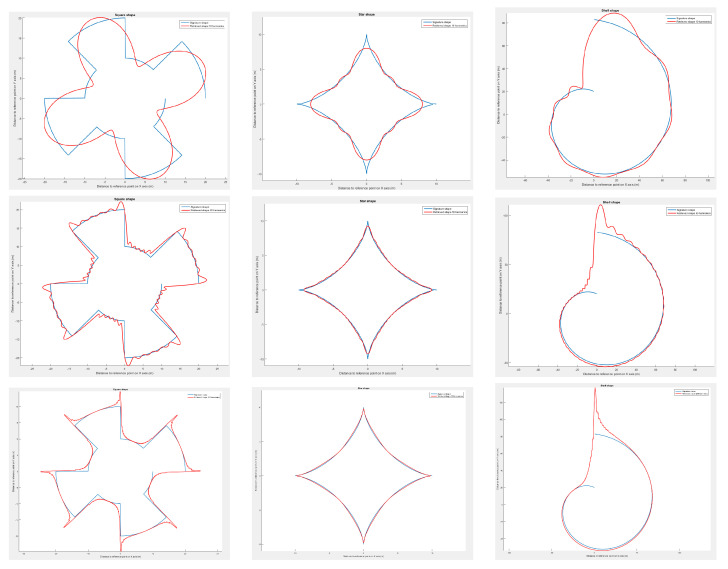
Shapes retrieved for various number of harmonics. From top to bottom, the number of harmonics equals 3,5,10,50, and 250.

It seems that the method using a polar FD lacks efficiency and robustness on shapes with high variations. We noticed the same phenomenon on the square signal and shell shapes even with large number of harmonics (see Figure A3 in Appendix A). Nonetheless, the non-continuity of the derivative seems to be handled flawlessly.

**Figure 13:**
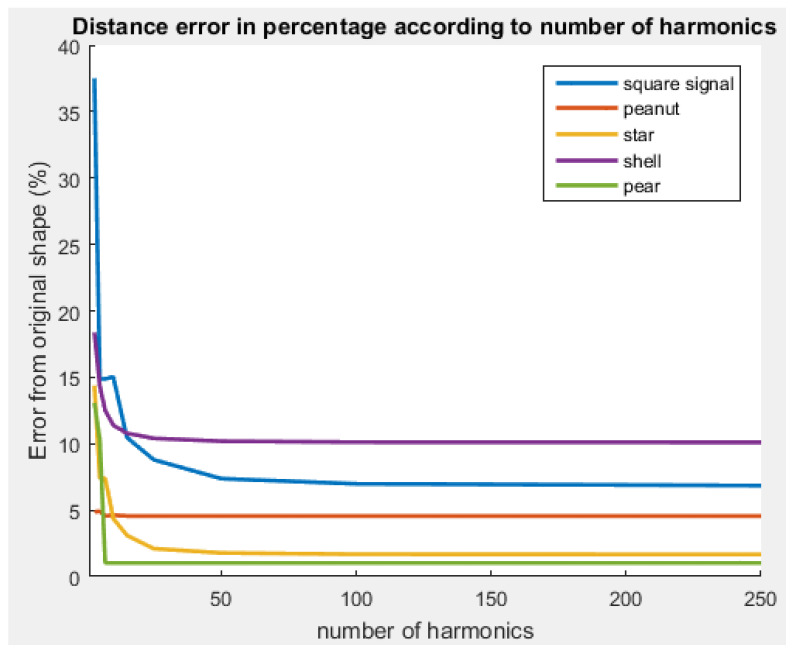
Evolution of the retrieval error according to the number of harmonics (based on Table A3 in Appendix A).

When varying the number of harmonics, the shape retrieval method does not behave the same on the whole set of shapes. In Figure 13, the only shape for which the error continuously increases with the reduction of harmonics is the shell. For the other shapes, an increase or decrease in the number of steps reduces the retrieval accuracy.

The increase in the error at certain specific steps provides clear evidence of the presence of null harmonics for certain shapes. Depending on the ease with which the shape can be interpreted with fewer harmonics, it is possible to reduce the number of harmonics in order to limit the amount of information required by the drones.

As an example, we deleted all harmonics with a radius lower than $1\times 10^{-3}$ prior to depiction by the FD. The resulting retrieval error is illustrated in Table 1.

Even when reducing the number of harmonics, the proposed method performs as if there were all 250 harmonics. Interestingly, the pear shape had only 2 harmonics after reduction. This means that only six values (2× (frequency, amplitude, phase)) need to be sent to the drones in order for them to have the full shape depiction. Instead of sending a precomputed (*x*, *y*, *z*) coordinate each time the drone needs to move along the shape, it now requires only a bearing angle (β) after having sent the harmonics. Thus, for the pear shape, if a drone needs to move around it more than twice, the amount of data that needs to be exchanged is lower with our method.

**Figure 14:**
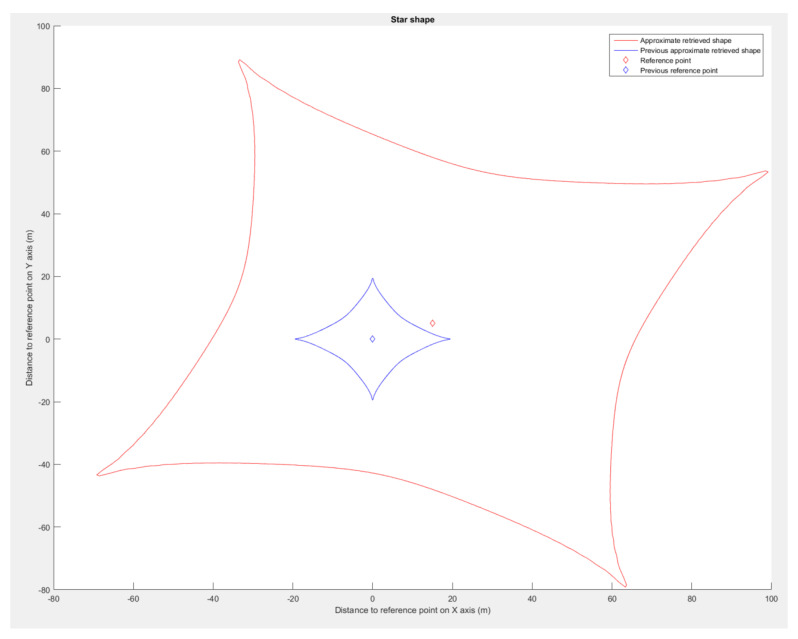
Astroidal shape transformation from previous parameters to new ones: scale = 100, rotation = π/3, reference point = (15,5).

Next, we visualized the shape retrieval process with different transformations applied (translation, rotation, and resizing). As a result, we were able to dynamically change the shapes without deformation or loss of continuity (Figure 14).

In this way, dynamic formation control can be performed without recomputing the harmonics or resampling the initial set of points fed to the DFT. The results prove that our method saves computation time when applying transformations on the same shape compared to standard method of point control of each drone, which needs to compute new coordinate for the drones each time.

Using the proposed method, light shows using drones to depict moving objects no longer require multiple computations to animate the represented shape.

**Figure 15:**
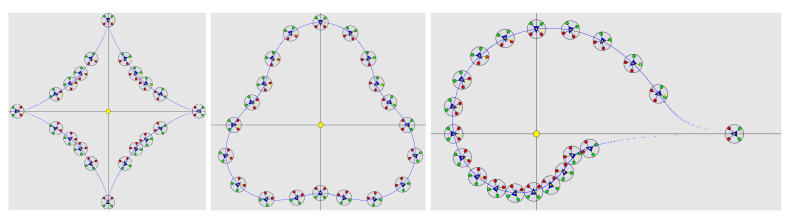
Simulation of astroidal, pear, and shell shape with twenty drones.

We used Processing [27] to simulate the formation control of twenty drones on the astroidal, pear, and shell shapes (Figure 15). UAVs were able to navigate to their self-computed positions without collision. They were able to form an angular distribution around the shape with only the shape’s harmonics. The angular distribution seems less relevant in case of asymmetrical and non-centered formations, for which a notion of curvilinear distance should be used instead.

**Figure 16:**
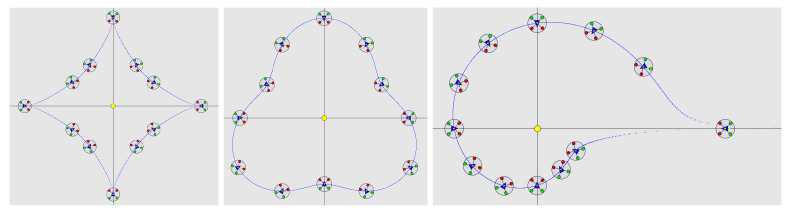
Simulation of astroidal, pear, and shell shapes with twelve drones after removing eight drones from the initial formation of twenty drones.

When we removed eight drones from the simulation, the drones were able to autonomously manage the resulting rearrangement (Figure 16).

**Figure 17:**
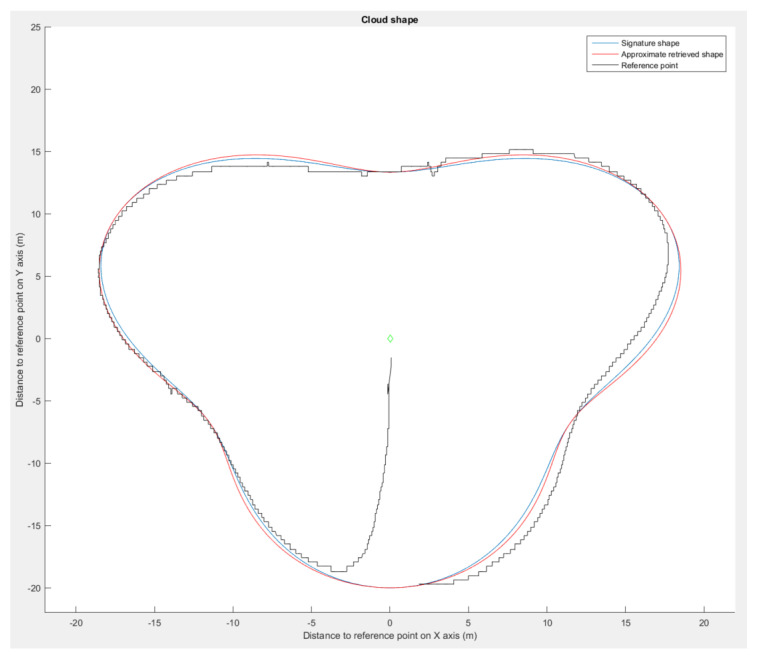
Pear shape simulation results, showing the signature shape (blue), approximated shape (red), and drone’s path during the simulation (black).

**Figure 18:**
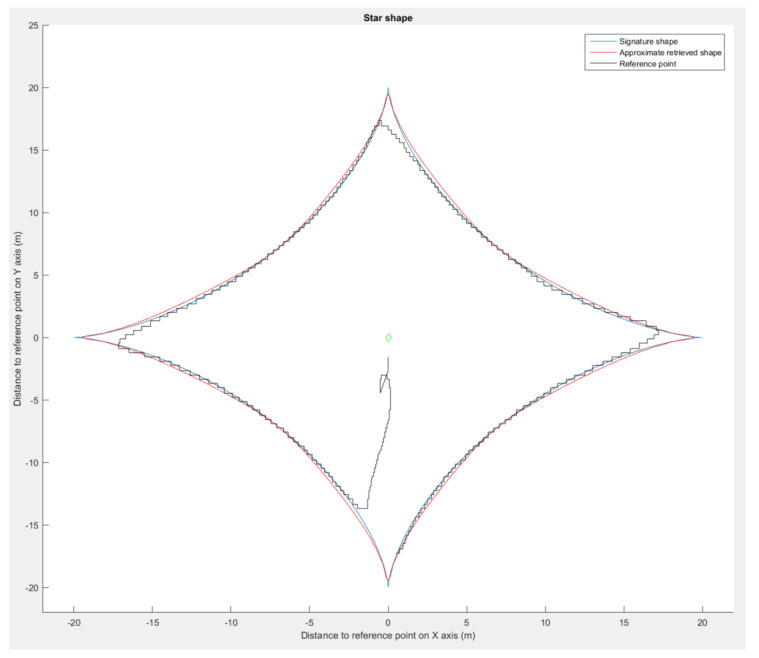
Astroidal shape simulation, showing the signature shape (blue), approximated shape (red), and drone’s path during the simulation (black).

Using the presented flight simulation setup, we made one drone navigate around the astroidal and pear shape. Examination of the data collected during the simulation (Figure 17 and Figure 18) validates the method.

**Figure 19:**
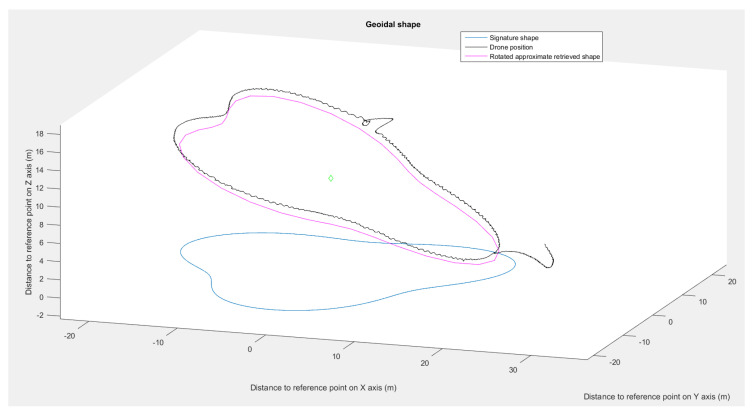
Pear shape simulation, showing the signature shape (blue), rotated approximated shape (pink), and drone’s path during the simulation (black).

The drone maintained satisfying placements even with spatial rotations (Figure 19), with positional deviations from the approximated shape of less than 10%. The deviations were caused by the control law used by the drone and inaccuracy of the simulated GPS. For the star shape (Figure 18), the drone showed signs of delay in following the bearing angle instructions in the cardinal directions, which entailed an increase in the positioning error. The retrieval method returned an approximated shape with a deviation lower than 2% in each run. These results validate the shape interpretation by the simulated drone even in with spatial rotations.

**Figure 20:**
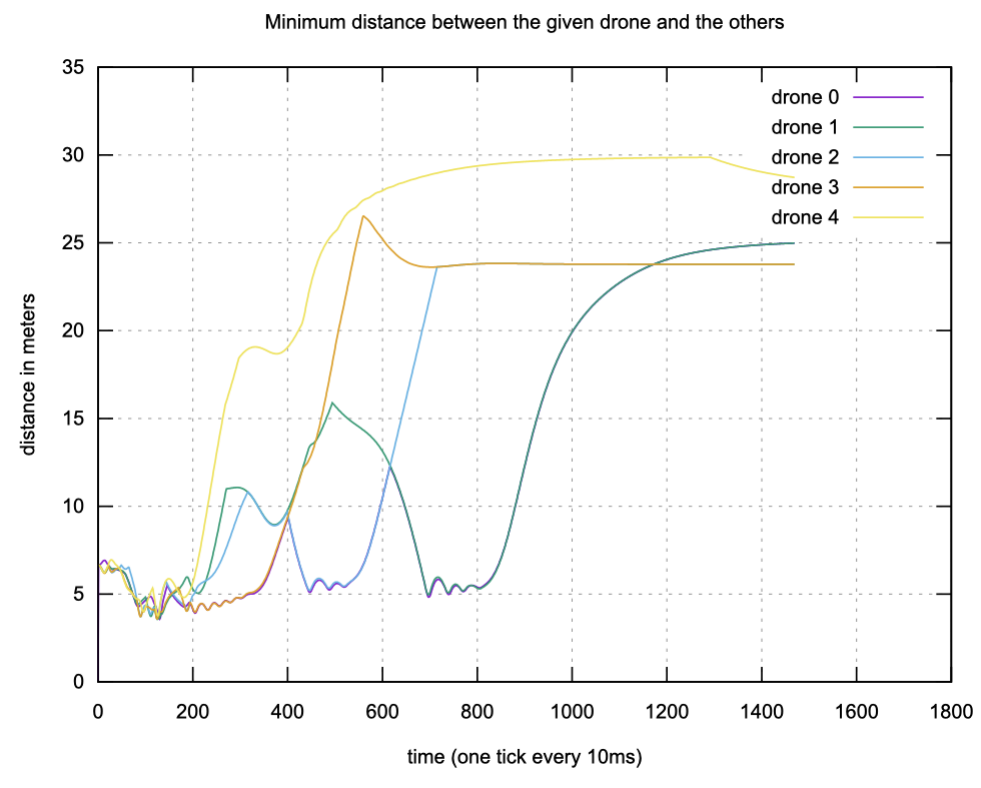
Minimum distance between drones during flight in the simulation. After take off, the drones move to their final positions. During the first two seconds, while the drones remain close to each other (the distance between them is less than 6 m), they never collide. Afterwards, they move further from one another. During the interval from 4 s to 6 s, drones 0 and 2 are moving closer, allowing the effect of the collision avoidance mechanism described in Section 4.1.4 to be observed. The same phenomenon can be observed for the period from 7 s to 9 s, during which drones 0 and 1 are moving closer to each other without colliding.

In addition, the drones were able to avoid collisions during the simulations. Figure 20 shows the inter-drone distances during a run with five drones. With the distance threshold for avoidance activation (${\bar{D}}_{j}$) set to 6 m, the drones maintained a distance of 4.22 m even in the worst case.

### 5.4. Experiments

**Figure 21:**
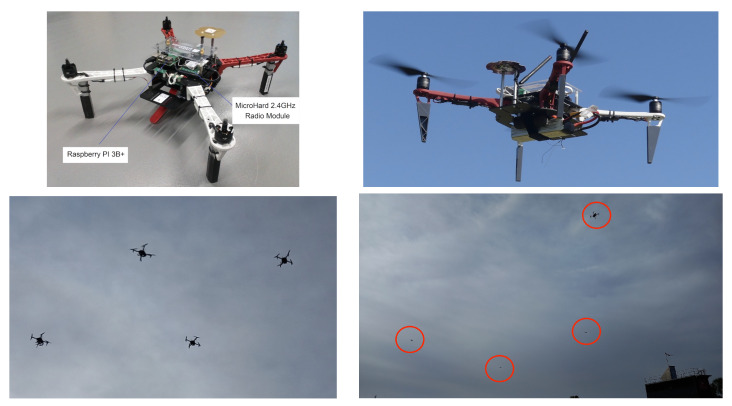
Our experimental plaform consisting of DJI F450 drones. The upper left-hand picture shows the computing devices (MiniSim and Raspberry PI), which were the same those used for the simulation (Figure 10). The images at the bottom were taken during the experiments. The red circles in the bottom left-hand image indicate the drones’ positions.

Experiments were been carried out using our home made drones (Figure 21), based on the frame kit F450 proposed by DJI (the well-known drone manufacturer) and equipped with a flight controller and an API designed by Squadrone Systems [26].

The flight controller and API were identical to those used in the Minisim Hardware-in-the-Loop simulators (Figure 10). The Fourier descriptors and control laws were implemented in C++ language on the Raspberry PI 3B+ embedded computers. Following our successful simulations, it was possible to download the same C++ code into the UAVs. The UAVs communicated with the ground station via MicroHard 2.4 GHz radio modules.

**Figure 22:**
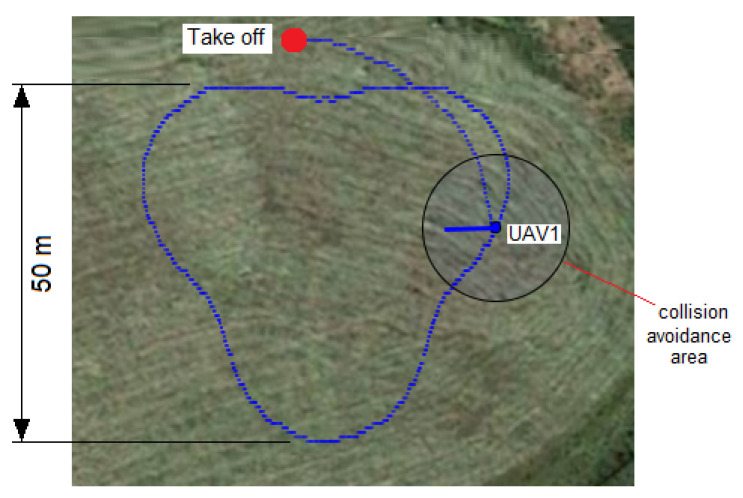
Pear shape followed by the UAV during the real experiment. The reference point and shape description were provided as the parameters of the method before the experiment. After take off, the drone successfully followed the shape while pointing in the direction of the reference point at each moment.

The goal of the experiments was to check whether one UAV was able to follow the contours of a particular shape described by means of Fourier descriptors. The experimental procedure consisted of choosing a shape and its characteristics (dimensions, inclination, etc.) and programming the increase of $\beta_{1}$ between 0 and $360^{\circ }$ in small steps of about 2${}^{\circ }$/s. Calculation of the values of $d_{\beta_{1}}$ and $\gamma_{1}$ was carried out using the Fourier descriptors method. An inclined pear shape ($10^{\circ }$, 50 m) was chosen for the experiments. We registered the GPS coordinates during the flight in order to represent the trajectory followed by the UAV (Figure 22).

**Figure 23:**
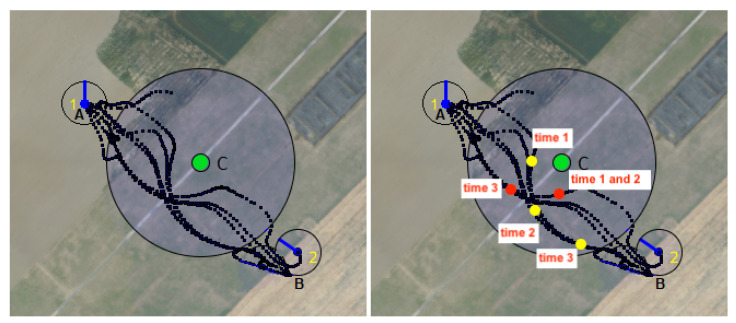
Collision avoidance of drones with obstacles and with each other. Two drones were programmed to move from point A to point B, with the first drone, represented by a yellow dot) starting at point A and the second (red dot) starting at point B. In the middle of the path between A and B is a static obstacle, C. The black dots correspond to the coordinates of each drone during their back and forth movement, positions that were logged during our real flight experiments. During the flight we can distinguish three periods. During the first period, node are moving toward their target. At time 1 they get close to the obstacle. Then, at time 2, due to the collision avoidance process, only one drone (the yellow one) is advancing toward its target, preventing the second one (red dot) to do the same and forcing it to remain at the same position. Finally during the last period (time 3), red drone has enough room to advance toward its target.

To validate the collision avoidance method, we carried out an experiment in which two UAVs flew to two opposite points (A and B) while avoiding a central point (C). In this experiment, the first UAV is initially at point A and the second is at point B. The two UAVs repeat this operation in a loop until the operator sends them the order to land. Taking disturbances (wind, etc.) into account, the UAVs can avoid each other by moving right, left, up, or down. Both UAVs are able to send their information (GPS coordinates, velocities, etc.) through the communication network via UDP multicast protocol and threads. We registered the GPS coordinates during the flight in order to represent the trajectory followed by the UAVs (Figure 23 (left image)).

In the right-hand image, the red and yellow dots illustrate the way in which the drones were able to avoid collisions with static obstacles and with other drones, respectively. At time stamp 1, the drones avoid collision with the obstacle and with each other. The first drone (the red dot) randomly changes its position while moving toward its destination (time 2). Its movement opsens up the space and allows the other drone (the yellow dot) to move towards its own destination (time 3).

## 6. Conclusions and Perspectives

In the aftermath of the industrial accident at Lubrizol in Normandy, France, which occurred in 2019, numerous questions were raised regarding the potential assistance drones could have provided in supporting the efforts of the firefighting teams. It became evident that a multi-UAV system could potentially provide a comprehensive 360 degree view of the operational theater, provided that a way could be found to offer such coverage. To achieve this objective, considering the source of the fire’s origin, several challenges must be overcome: (i) precisely describing the area to of interest; (ii) allowing changes in the scale and orientation of the shape surrounding this area; and (iii) ensuring continuous 360 degree vision by allowing the group’s composition to be modified as drones depart from the formation and new drones arrive.

In this context, we have proposed a method for multi-UAV formation control. This method relies on Fourier descriptors and transform along with decentralized algorithms for allocating the positions of drones within the group. The decentralized algorithms for allocating bearing angles have only been tested through simulation. However, the restricted number of exchanged messages (with respect to the number of drones) required to reach a consensus provides optimism for their performance in real-world settings. For the description of the surrounding shape, polar signature functions were tested as formations and several transformations have been applied for the astroidal shape. In addition, continuous rotation around the shape border was tested for a UAV. The proposed method demonstrates great accuracy in terms of shape reproduction even with transformations. The simulated multi-UAV system further validates the method, showing satisfying placements. Further experiments and developments could be achieved by using virtual actions to smooth the UAVs’ movements and prevent deadlock situations. An improvement in the robustness of shape retrieval should be made to prevent border effects caused by discontinuous signature functions. Finally, this system could be used to approximately model objects by referencing points one-by-one at its border.

## Figures and Tables

**Table 1:** Retrieval distance relative error with reduced harmonics.

Shape	nb.har.	Moy.	Max.	Var.	%
Sqr.Signal	63	0.068459	1.4309	0.025236	6.85
Peanut	18	0.045551	0.10431	0.0032394	4.56
Star	32	0.016736	0.042251	0.00040562	1.67
Shell	250	0.10098	5.2529	0.13764	10.1
Pear	2	0.010257	0.024512	0.00016158	1.03

**Figure A1:**
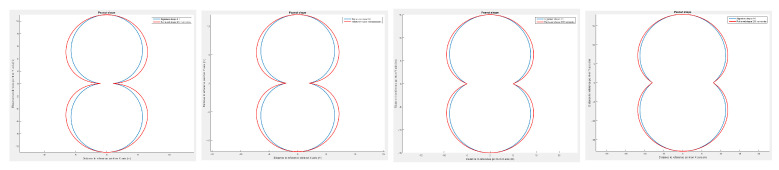
Peanut shape concave factor (*n*) variation. From left to right, the figures were obtained using values of *n* equal to 0.1, 0.2, 0.5 and 0.8.

**Figure A2:**
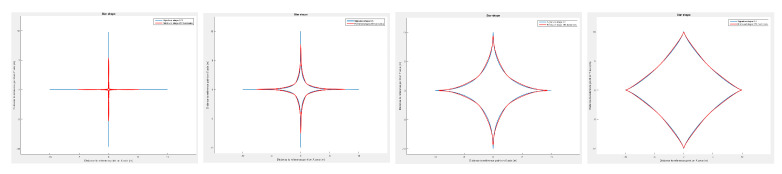
Star shape concave factor (*n*) variation. From left to right, the figures were obtained using values of *n* equal to 0.15, 0.3, 0.5 and 0.8.

## Data Availability

Data are contained within the article and Supplementary Material.

## Supplementary Materials

A video of the simulation of five drones describing the pear shape can be downloaded at https://www.mdpi.com/article/10.3390/s23239553/s1.

## Appendix A

### Appendix A.1. Data Tables Resulting from Shape Retrieval Characterization

**Table A1:** Retrieved distance error for the peanut shape (concave shape) from Figure A1.

	Absolute Error			Relative Error			
**Coeff.**	**Moy.**	**Max.**	**Var.**	**Moy.**	**Max.**	**Var.**	**%**
0.1	0.72722	1.448	0.77411	0.15035	0.43336	0.041047	15.04
0.2	0.62662	1.2525	0.57414	0.10041	0.25381	0.016967	10.04
0.3	0.5518	1.1058	0.4445	0.073662	0.17531	0.0087876	7.37
0.4	0.49356	0.99175	0.35506	0.056942	0.13086	0.0051283	5.69
0.5	0.44679	0.89969	0.29053	0.045586	0.10247	0.003234	4.56
0.6	0.40834	0.82449	0.24235	0.037443	0.08294	0.002156	3.74
0.7	0.37613	0.7612	0.20539	0.031369	0.068797	0.0014996	3.14
0.8	0.34874	0.70763	0.17638	0.026701	0.058123	0.0010788	2.67

**Table A2:** Retrieved distance error for the star shape (convex shape) from Figure A2.

	Absolute Error			Relative Error			
**Coeff.**	**Moy.**	**Max.**	**Var.**	**Moy.**	**Max.**	**Var.**	**%**
0.15	0.16281	4.7764	0.3243	0.4389	6.8007	0.70728	43.89
0.2	0.15252	4.2607	0.22692	0.15079	2.0895	0.066893	15.08
0.3	0.15976	2.7085	0.10127	0.06572	0.54591	0.0077634	6.57
0.4	0.1581	1.5494	0.05486	0.042037	0.2312	0.0027172	4.20
0.5	0.14267	0.94335	0.034822	0.029123	0.11666	0.0012425	2.91
0.6	0.12093	0.56702	0.022815	0.020734	0.0637	0.00062064	2.07
0.7	0.098478	0.33963	0.014614	0.014942	0.036249	0.00032156	1.49
0.8	0.077903	0.20364	0.009047	0.010811	0.024793	0.00016909	1.08

**Table A3:** Evolution of the retrieved distance relative error (in percentage) according to the number of harmonics. These results are illustrated by Figure A3 for a subset of these numbers of harmonics. An improvement in the description of the pattern can be observed when the number of harmonics increases.

nb.har.	Sqr.Signal	Peanut	Star	Shell	Pear
3	37.50	4.88	14.38	18.37	13.08
5	14.88	4.91	7.39	14.33	10.3
7	14.88	4.56	7.39	12.5	1.03
10	15.03	4.62	4.36	11.39	1.03
15	10.47	4.56	3.11	10.79	1.03
25	8.79	4.56	2.1	10.4	1.03
50	7.37	4.56	1.78	10.19	1.03
100	6.98	4.56	1.68	10.12	1.03
250	6.85	4.56	1.67	10.1	1.03

### Appendix A.2. Algorithms for Known Number of UAVs

**Algorithm A1** Algorithm executed by each drone for known number of drones (*N*).
1: create and initialize Positions[N] 2: myPosition← random(*N*) 3: change ←true 4: **while** ∃i, Positions[i]=false **do** 5: **if** change **then** 6: Positions[myPosition]←true 7: broadcast Positions[] and myPosition 8: change ←false 9: **else** 10: **if** receivedPositions[] and peerPosition have been received **then** 11: **if** Positions[]≠receivedPositions[] **then** 12: merge Positions[] and receivedPositions[] 13: /* if receivedPosition[j] is true then Positions[j] becomes true */ 14: change ←true 15: **end if** 16: **if** myPosition=peerPosition **then** /* conflict */ 17: myPosition←random(N) such that Positions[myPosition]=false 18: Positions[myPosition]←true 19: change ←true 20: **end if** 21: **end if** 22: **end if** 23: **end while** 24: broadcast Positions[] and myPosition 25: compute the angle then the destination point 26: move to the destination point

### Appendix A.3. Algorithms for Dynamic Number of UAVs

**Algorithm A2** Algorithm executed by each drone for changing number of drones.
1: mailbox ←∅ 2: N←1 3: create and initialize Positions[1] 4: myPosition←1 5: change ←true 6: broadcast (1,Positions,JOIN) 7: newToTheGroup ←true 8: **while** mission is not finished **do** 9: **if** mailbox has some messages **then** 10: /* messages are received into the mailbox by another process asynchronously */ 11: change ←false 12: **while** mailbox has some messages **do** 13: change ← Process Messages (Algorithm A3) 14: **end while** 15: **else** 16: **if** all cells of Positions[] are true **then** 17: compute bearing angle 18: **else** 19: change ←true 20: **end if** 21: **end if** 22: **if** change **then** 23: broadcast (myPosition,Positions[],UPDATE) 24: change ←false 25: **end if** 26: **end while** 27: Positions[myPosition]←false 28: broadcast (myPosition,Positions[],LEAVE)

**Algorithm A3** Process messages.
1: read next message: uavPosition, receivedPositions, msgType 2: **if** msgType is JOIN **then** 3: N←N+1 4: Positions[N]←false 5: change ←true 6: **end if** 7: **if** msgType is LEAVE **then** 8: N←N−1 9: **if** uavPosition<myPosition **then** 10: myPosition←myPosition−− 11: update Positions[] 12: **end if** 13: change ←true 14: **end if** 15: **if** msgType is UPDATE **then** 16: **if** newToTheGroup AND size of receivedPositions[]>1 **then** 17: update *N* 18: update Positions[] 19: change ←true 20: **else** 21: **if** myPosition=uavPosition **then** 22: myPosition← find a random free position 23: update Positions[] 24: change ←true 25: **else** 26: update Positions[] 27: **if** receivedPositions[myPosition]=false **then** 28: change ←true 29: **end if** 30: **end if** 31: **end if** 32: **end if** 33: newToTheGroup ←false 34: return change
